# Acute Tibial osteomyelitis caused by intraosseous access during initial resuscitation: a case report and literature review

**DOI:** 10.1186/s12879-018-3577-8

**Published:** 2018-12-17

**Authors:** Thomas Chalopin, Adrien Lemaignen, Antoine Guillon, Arnaud Geffray, Gaelle Derot, Olivier Bahuaud, Charles Agout, Philippe Rosset, Claire Castellier, Gonzague De Pinieux, Anne-Sophie Valentin, Louis Bernard, Frederic Bastides, Centre De Référence Des Infections Ostéo-Articulaires Du Grand-Ouest (CRIOGO) Study Team

**Affiliations:** 1 Department of Internal Medicine and Infectious Diseases, University Hospital of Tours, Hospital Bretonneau, Tours, France; 2 François Rabelais University, Tours, France; 3 Department of Intensive Care Unit, University Hospital of Tours, Tours, France; 4 Department of Medical Imaging, University Hospital of Tours, Tours, France; 5 Department of Orthopedic Surgery, University Hospital of Tours, Tours, France; 6 Department of Anatomopathology, University Hospital of Tours, Tours, France; 7 Bacteriological Laboratory, University Hospital of Tours, Tours, France; 8 2 boulevard Tonnellé, 37044 Tours, Cedex 9 France

**Keywords:** Acute osteomyelitis, Intra-osseous access, Resuscitation, *Staphylococcus aureus*, Antibiotics

## Abstract

**Background:**

Intra-osseous (IO) access is recommended in cases of pre-hospital emergency or resuscitation when intravascular (IV) route is difficult or impossible. Despite recent improvement in IO devices and increasing indications, it remains rarely used in practice. Various complications have been reported but are uncommon.

**Case presentation:**

We report a case of massive acute tibial osteomyelitis in an adult male three months after an IO catheter insertion for emergency drug infusion. We review the literature on association between IO access and acute osteomyelitis in children and adults.

**Conclusions:**

Emergency-care givers and radiologists should be informed about this infrequent complication in order to make early diagnosis and initiate adequate antibiotic therapy.

## Background

Intraosseous (IO) access is considered as an effective route in adults requiring emergency administration of fluids or medication for initial resuscitation [1]. IO infusion is an important and safe alternative to the intravenous route in cases of difficult vascular access due to obesity, edema, or exhausted venous access in special populations (e.g drug-addicts). Despite improvements in IO devices and prehospital management of cardio-pulmonary resuscitation, IO infusion is not commonly used in clinical practice [2, 3]. Complications after IO access are uncommon. However, extravasation, air embolism or skin abscesses have been reported. Osteomyelitis occurs in less than 1% [4, 5] with a very small number of cases reported, to the best of our knowledge [6–10]. We report an unusual case of massive acute tibial osteomyelitis in an adult, three months after an IO infusion used in an initial resuscitation at home. We proposed to review the cases published in the English language literature with paying attention particularly to risk factors, diagnosis, treatment and outcome, to contribute to the description and management of this condition.

## Case presentation

A forty-year-old psychotic and intravenous-drug-addicted Caucasian male was cared by prehospital service for coma due to drugs overdose. In this emergency situation, without any intravenous access available, an IO device (EZ-IO™; Teleflex Medical, Research Triangle Park, NC, USA.) was promptly inserted by the emergency medical technician (EMS) on scene in the upper portion of the left tibia to administer therapeutics and initiate mechanical ventilation. Hospitalization in the intensive care unit of the University Hospital of Tours (France) with close monitoring lasted three days. Several attempts to establish another IV catheter were unsuccessful and no central catheter was used as the need for infusion was expected to be short due to a rapid clinical improvement. The IO catheter was removed at Day 1, with report of local inflammation around the insertion site. An erysipelas was diagnosed. Treatment with oral amoxicillin-clavulanic acid (1gx3/day) was introduced. The patient reported psychiatric problems with schizophrenia, multiple intravenous-drug intoxications with coma, and regular cocaine and heroin use. He left the hospital against medical advice three days after IO device removal.

**Fig. 1:**
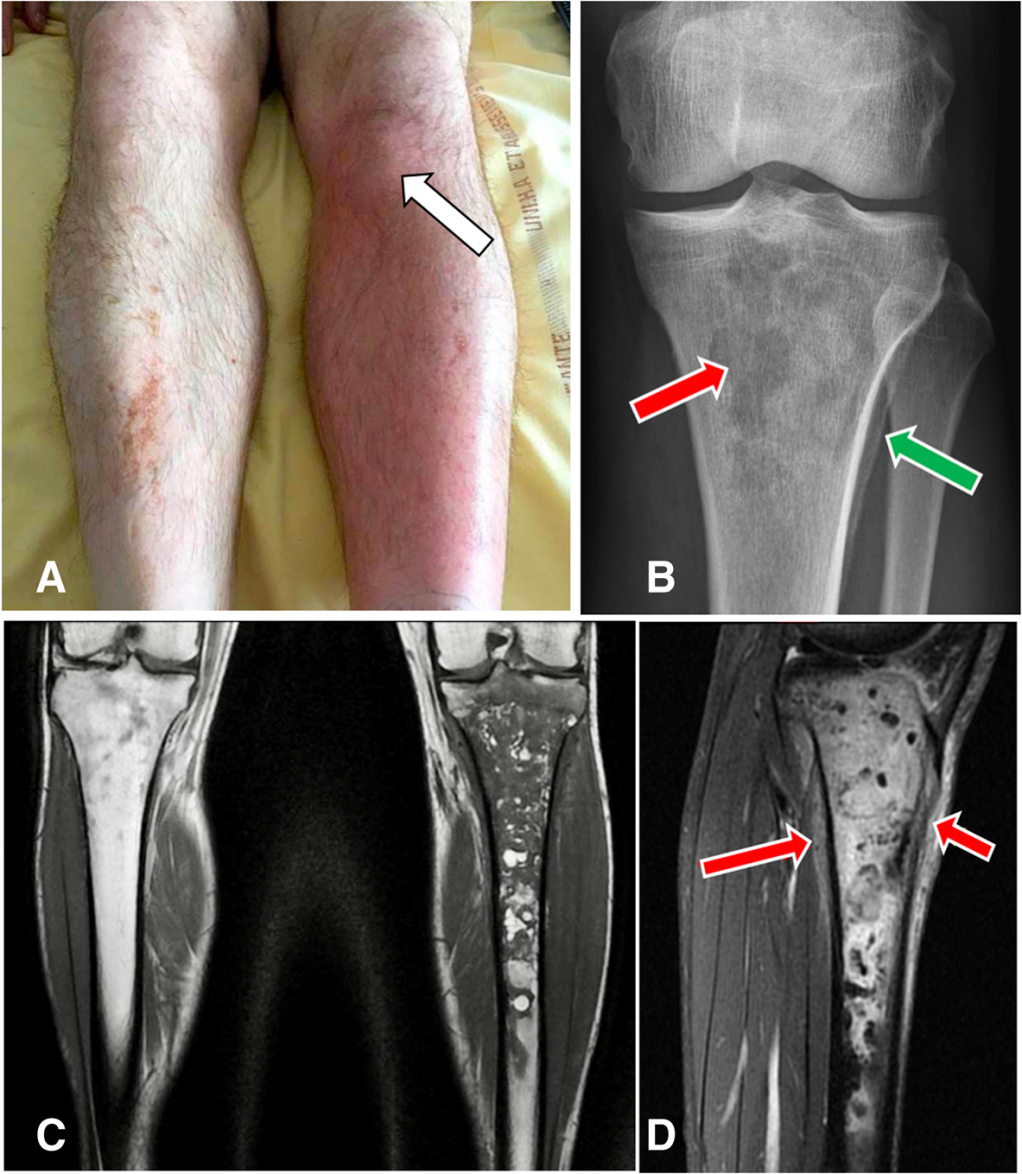
**a** Image of the left tibia, well-defined area with redness, warmth and swelling over his left proximal tibia corresponding to the site of the intraosseous injection (arrow). The contralateral leg is normal. **b** X-rays of left proximal tibia in front view with multiple ill-defined lytic lesion in the metaphysis and the epiphysis and geographic pattern (red arrow) and also proximal tibial blurred periosteal appositions (green arrow). **c** Coronal T1-weighted section with massive epiphyso-metaphyseal hypoT1 intra-medullary bone edema extended to the diaphysis up to the middle third of the leg. **d** Sagittal fat-sat T2–weighted section with significant hyperT2 infiltration of soft tissues (arrows)

Three months later, he asked for a consultation in the same hospital because of fever and bone pain in the left leg and was hospitalized again. Other complaints were chills and inability to walk normally and to bear weight on his left leg. Redness, warmth, point tenderness and swelling on the site of the IO access were present (Fig. 1a, arrow). He was afebrile, without hemodynamical instability. Laboratory results were only significant for leukocytosis at 12.4.10^9^/L and C reactive protein at 51.2 mg/l. Blood cultures were negative. Routine radiographs revealed an ill-defined osteolysis of the metaphysis and the epiphysis with a condensed area and blurred periosteal appositions (Fig. 1b). The magnetic resonance showed an important marrow edema with T1-weighted hyposignal (Fig. 1c) and fat-saturated-T2-weighted hypersignal (Fig. 1d) extending in the left tibia, measuring twenty-one centimeters. Soft tissues were infiltrated. No abscess was visualized but the radiologist could not achieve gadolinium injection because IV access was lacking. MR imaging was compatible with the diagnosis of osteomyelitis.

An open biopsy of the left tibia revealed Gram positive cocci with focal signs of acute osteomyelitis, bone remodeling and marrow fibrosis containing polymorphic inflammatory infiltrate comprising neutrophils, foamy macrophages, lymphocytes and plasma cells. Treatment with piperacillin-tazobactam and vancomycin was initiated. Culture from the surgical site grew methicillin-susceptible *Staphylococcus aureus* (MSSA). An oral switch with levofloxacin 750 mg/day and rifampin 900 mg/day for six weeks was introduced. A favorable outcome was noted eighteen months later.

## Discussion

IO infusion provides access to a non-collapsible venous complex, enabling administration of resuscitation drug therapy to start treatment of shock, cardiac arrest or in severe trauma. In adults, the European Resuscitation Council Guidelines for Resuscitation established in 2015 that IO route is required in emergency situations whenever peripheral access cannot be achieved: it can be used for infusion, drug administration and blood samples [11]. Two prospective trials in children and adults, consolidated by several studies, documented that IO access was safe and effective for initial resuscitation cases [12–16].

In practice, IO-access is rarely used despite many advents in IO insertion devices making the procedure easier and faster [3, 17]. In 2012 in France, 29% of practitioners (intensivists, EMS, anesthetists) have used an IO kit in real-life. Concerning training, 55% of them have been trained to IO procedure (83% of EMS and 33% of intensivists) [18]. Currently, several IO insertion devices are approved by the Food & Drug Administration (FDA) [19]. Different insertion sites have been evaluated with three possible locations: the proximal tibia, the distal tibia and the proximal humerus [20]. The most frequently used is the proximal tibia, located 2 cm below the tibial tuberosity and 1 to 2 cm medial in the middle of the flat bone surface as in our patient [21].

The procedure of IO access must be performed under strictly sterile conditions. Even if multiple studies proved IO access to be safe and effective compared to IV route, various complications have been reported [1, 22, 23]. Two studies concluded that fluid extravasation is the most common complication (12%) following by skin abscesses, cellulitis or embolism [4, 5].

Acute osteomyelitis is an inflammatory process in bone and bone marrow, most often caused by pyogenic bacteria, with different pathogenesis, either haematogenously-acquired, or associated with peripheral vascular disease like diabetes, or with contiguous-focus infection as in our case. Today, the spectrum of osteomyelitis is changing. It occurs most frequently from contiguous-focus spread after an open fracture, reconstructive surgery, or with a direct inoculation from trauma as IO access. Therefore, we performed an extensive literature search of databases for articles published up to 1997, to correctly report the osteomyelitis incidence with new IO devices. We found only two cases in the literature reporting an acute osteomyelitis in an adult after IO infusion, other cases occurred in children where IO access is more frequent (Table 1). In published studies with a large cohort, osteomyelitis occurs in less than 1% of patients (children and adults) and thus is the most unusual late complication reported due to direct inoculation. In a retrospective, online questionnaire-based investigational study, in 1.802 cases of IO use, the rate of osteomyelitis is 0.4%, corresponding to seven patients [23]. This rate is very close to those reported by *Rosetti* et al.*,* (0.6%) or *Leidel* et al.*,* (0.6%) [5, 12]. Published data suggest that osteomyelitis is considered as a serious late complication to IO infusion, with a very low rate, with clinical and bacteriological heterogeneity. This is the first case of IO-access acute osteomyelitis reported in our institution with the use of around 40 IO-devices each year.

**Table 1:** Published cases of acute osteomyelitis caused by intraosseous devices in children and adults in literature

Reference	Sex, age	Interval days^a^	Predisposing factor	Patient condition at the time of IO insertion	Site	Duration of IO device	Culture	Treatment	Outcome
[6]	Male, 5 months	10 days	–	Initial resuscitation	Proximal tibia	*Not cited*	*Candida albicans*	Fluconazole	Recovered
[7]	Male, 62 years	6 months	MGUS, diabetes	Initial resuscitation	Proximal tibia	*Not cited*	*MRSA*	Vancomycin	Recovered
[8]	Male, 14 months	3 days	-	Initial resuscitation	Bilateral distal femur	*Up to 24 h*	*Escherichia coli, Enterobacter cloacae*	Unknown	Unknown
[9]	Male, 3 months	24 h	Spastic tetraplegia	Initial resuscitation IO access no longer functional	Proximal tibia	*2 h*	*Acinebacter baumannii*	Amoxycillin, netilmicin, ceftazidime	Died
[10]	Male, 29 years	6 weeks	Drug-addict	Initial resuscitation No peripheral IV access	Proximal tibia	*1 h*	*Escherichia coli, MSSA*	Ceftriaxone, metronidazole, ertapenem	Recovered
Our report	Male, 40 years	3 months	Drug-addict	Initial resuscitation No peripheral IV access	Proximal tibia	*24 h*	*MSSA*	Levofloxacin, rifampicin	Recovered

*MRSA* methicillin-resistant *Staphylococcus aureus, MSSA* methicillin-susceptible *Staphylococcus aureus, IO* intraosseous, *MGUS* monoclonal gammopathy of undetermined significance, ^*a*^Interval between IO insertion device and admission to hospital

Our case demonstrated a massive delayed osteomyelitis in a comorbid drug-addict patient who is predisposed to cutaneous infection, and with a frequent indolent course reported in osteomyelitis secondary to a contiguous focus of infection. Even if erysipelas was initially suspected and treated, treatment was not taken entirely and osteomyelitis was rapidly evoked three months later after clinical examination and X-Ray. This data emphasizes that complete and early treatment of the erysipelas might prevent the development of subsequent osteomyelitis in this situation. Rapid management is required to avoid more severe complications such as chronic bone and articular dislocation. The key for successful management is early diagnosis, based on clinical examination, imaging procedures including conventional radiographs and MRI, bone sampling (open biopsy) for microbiological and pathological examination to enable targeted and long-lasting antibiotic strategy. *Staphylococcus aureus* is definitely the most frequent pathogen responsible for osteomyelitis in any age group, mainly methicillin-susceptible strains (MSSA), and it is responsible for up to 70–90% of confirmed cases [24, 25]. This infection requires prompt antibiotic therapy, initially IV and then switch to oral antibiotics. However, no consensus was found regarding the duration of antibiotic treatment, but some authors suggest the possibility of reducing the IV antibiotic duration to a few days and “step-down” to an oral antibiotic with targeted oral therapy [26, 27] for at least six weeks. In the future, development of novel and more effective strategies, such as the local delivery of antibiotics, biofilm disruptors or immunotherapy could improve therapeutic outcomes.

## Conclusion

In conclusion, intraosseous infusion is an effective alternative if IV access is not readily attainable for adults requiring urgent parenteral access for initial resuscitation. We reported here a potentially limb threatening acute tibial contiguous-focus osteomyelitis three months after intraosseous catheter insertion. One has to keep in mind that adverse events with low incidence rate can be easily underestimated. Thus, we believe that it is important to continue to report long-term infectious complications of this unusual infusion procedure as they are difficult to capture by conventional clinical trials.
